# Nutritional Mediators of Cellular Decline and Mitochondrial Dysfunction in Older Adults

**DOI:** 10.3390/geriatrics6020037

**Published:** 2021-04-06

**Authors:** Jack M. Guralnik, Jerome N. Feige, Anurag Singh, Roger A. Fielding

**Affiliations:** 1 Epidemiology & Public Health, University of Maryland School of Medicine, Baltimore, MD 21201, USA; jguralnik@som.umaryland.edu; 2 Nestlé Research, CH-1015 Lausanne, Switzerland; Jerome.Feige@rd.nestle.com; 3 Amazentis SA, CH-1015 Lausanne, Switzerland; asingh@amazentis.com; 4 Nutrition, Exercise Physiology, and Sarcopenia Laboratory, Jean Mayer USDA Human Nutrition Research Center on Aging, Tufts University, Boston, MA 02111, USA

**Keywords:** sarcopenia, muscle, mitochondria, nicotinamide riboside, urolithin A, age-associated cellular decline, accelerated aging and cellular decline, cellular nutrition, AACD

## Abstract

Aging is a primary risk factor for the progressive loss of function, disease onset, and increased vulnerability to negative health-related outcomes. These clinical manifestations arise in part from declines in mitochondrial, metabolic, and other processes considered to be hallmarks of aging. Collectively, these changes can be defined as age-associated cellular decline (AACD) and are often associated with fatigue, reduced strength, and low physical activity. This manuscript summarizes a recent Gerontological Society of America Annual Scientific Meeting symposium that explored mechanisms, clinical signs, and emerging cellular nutrition interventions for AACD. The session opened by highlighting results of an expert consensus that developed an initial framework to identify self-reported symptoms and observable signs of AACD in adults aged >50 years. Next, findings from the multi-ethnic molecular determinants of sarcopenia study were discussed, showing impaired mitochondrial bioenergetic capacity and NAD^+^ metabolism in skeletal muscle of older adults with sarcopenia. Lastly, recent clinical evidence was presented linking urolithin A, a natural mitophagy activator, to improved mitochondrial and cellular health. The virtual panel discussed how stimulation of mitochondrial function via biological pathways, such as mitophagy and NAD^+^ augmentation, could improve cellular function and muscle health, potentially impacting clinical signs of AACD and overall healthy aging.

## 1. Introduction

Geroscience has proposed that the underlying biological mechanisms of aging are central to the global increase in susceptibility to disease and disability that occurs with aging. Strong correlations have been revealed between biological mechanisms and some clinical phenotypes that are typical of aging, particularly declines in autophagy, mitochondrial dysfunction, and cellular senescence [1]. Studying the common cellular declines and modifiable signs and risks serves to identify promising targets for future interventions aimed at slowing aging and preventing age-related clinical conditions.

**Figure 1:**
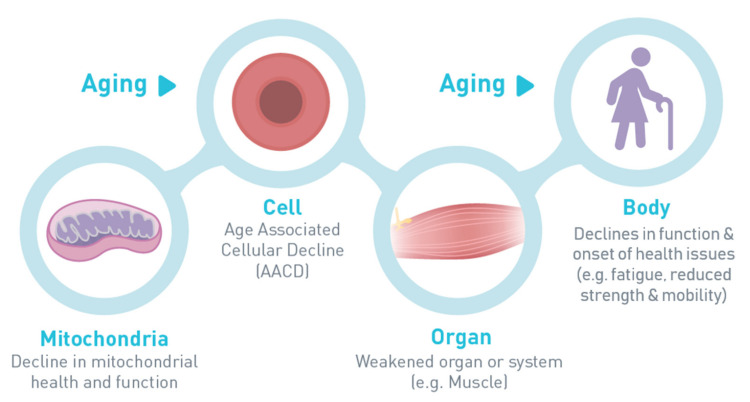
Mitochondrial origin of age-related decline.

Recently, experts convened at the virtual 2020 Annual Scientific Meeting of the Gerontological Society of America to review recent developments to target the origins of cellular decline, understand the modifiable signs and risks of accelerated aging and cellular decline, discuss data showing how mitochondrial dysfunction in older adults impairs muscle bioenergetics and function, and share emerging evidence on nutritional interventions to mitigate age-related mitochondrial dysfunction and improve health. As muscle tissues have a high abundance of easily accessible mitochondria and are of clinical significance to overall altered mitochondrial morphology, special attention was given to the common changes in skeletal muscle—from mechanisms to symptoms and performance (Figure 1)—tightly linked to well-being as adults grow older [2]. This proceedings manuscript captures the key points of the presentations and panel/audience discussion.

## 2. Early Detection of Age-Associated Cellular Decline: Report of an Expert Consensus (Dr. Jack M. Guralnik)

In a landmark article, López-Otín and colleagues [3] first proposed nine biological hallmarks of aging: genomic instability, telomere attrition, loss of proteostasis, deregulated nutrient sensing, altered intracellular communication, cellular senescence, stem cell exhaustion, epigenetic alterations, and mitochondrial dysfunction. Each of these nine hallmarks is considered to (1) occur during normal aging, (2) can accelerate the aging process, and (3) be potentially modifiable.

The medical model has traditionally focused on identifying and treating specific disease states and individual organ failure exhibited by older persons. This is related to but distinct from a more biologically driven approach of detecting cellular dysfunction, which often overlaps in different organs and predisposes the development of multiple age-related conditions. Moving beyond the traditional disease-specific focus to better promote healthy aging involves developing tools to better characterize the signs and symptoms of age-associated cellular decline (AACD) to facilitate early identification of those at risk of accelerated aging. To further this change in clinical approach, two interdisciplinary expert panels were recently convened to propose a novel framework to classify risk factors, early indicators, and clinical differentiators of AACD for the purpose of identifying signs, symptoms, and markers indicative of AACD [4].

A group of international experts with clinical and research expertise was convened through a virtual workshop for decision-making in this area. A consensus was established using the modified nominal group technique [5,6]. Briefly, the nominal group technique obtains views of experts on a given topic to bring about group consensus in a face-to-face setting. The preliminary theme and draft key principles were drafted by five experts from four different countries in the field of geriatrics. An extended group of thirteen experts from eleven different countries then reviewed and critically commented on the draft framework. Due to international travel and safety limitations imposed by the current COVID-19 pandemic, the face-to-face component was adapted to a virtual focus group setting [4]. Qualitative information was collected from experts on the following predetermined topics and questions:

- What are the early signs and symptoms of AACD?
- How could early age-associated decline be differentiated from other conditions with similar symptoms?
- What are the risk factors for AACD?
- Describe the profile of individuals at high risk of AACD?
- Which of the features of AACD are clinically relevant and important triggers for intervention? [4]

Table 1 highlights prioritized physical and environmental risk factors for AACD, presented in rank order. The main framework for identifying AACD is displayed in Table 2 [4]. Overall, the group defined the following modifiable signs and symptoms of AACD after middle age: poor exercise tolerance, a chronic condition, obesity, sedentary lifestyle, high exposure to smoke and/or alcohol, fatigue, memory complaints, low mood or motivation, and poor-quality sleep [4]. The clinical indicators (Table 2), if present, should be identified and treated, irrespective of age or disease status. Using the framework for early detection of cellular decline could be pivotal to a biology-driven and function-focused approach for managing AACD. Yet, several challenges may exist. Further research will characterize the various clinical indicators’ predictive power and help improve the tools for clinical practice. In addition, AACD should be differentiated from other conditions, and biomarkers could be viable for differential diagnostic applications in the future [4].

## 3. Markers of Altered Muscle Mitochondrial Bioenergetics in Older Adults with Sarcopenia and the Role of Nicotinamide Adenine Dinucleotide (Dr. Jerome N. Feige)

Skeletal muscle is important for overall health, and loss of muscle mass or strength is a powerful predictor of poor health outcomes [7]. A recent systematic review of population-based studies demonstrated the overall worldwide prevalence of sarcopenia to be approximately 10% in men and women aged ≥60 years [8]. To better develop molecular markers for the identification of sarcopenia, the multi-ethnic molecular determinants of sarcopenia (MEMOSA) study recently compared genome-wide transcriptomic profiles of skeletal muscle biopsies from men aged ≥65 years diagnosed with sarcopenia with age-matched controls [9]. The study identified for the first time that the muscle of individuals with sarcopenia had reduced activity of the key energy-producing pathways and a decrease in the activity of all five mitochondrial respiratory complexes in the energy production pathway deemed critical for maintaining muscle strength and function. Mitochondrial dysfunction was the strongest molecular signature of sarcopenia, replicated across three distinct ethnic cohorts from populations in the United Kingdom, Jamaica, and Singapore. Individuals with sarcopenia have reproducibly demonstrated a strong transcriptional signature of mitochondrial bioenergetic dysfunction in skeletal muscle with low signaling of the two transcriptional regulators, estrogen-related receptor α and its coactivator peroxisome proliferator-activated receptor γ coactivator 1α, which has been shown to negatively impact oxidative phosphorylation. These demonstrated changes translate functionally to fewer mitochondria, reduced mitochondrial respiratory complex expression and activity, and low nicotinamide adenine dinucleotide (NAD^+^) levels through perturbed NAD^+^ biosynthesis and salvage in sarcopenic muscle cells [9].

A reduction in NAD^+^ can lead to a loss of mitochondrial homeostasis [10]. NAD^+^ levels have been shown to be downregulated by approximately 30% among individuals with sarcopenia [9]. Mitochondrial dysfunction can be induced by multiple factors, including energy-dense diets and aging through depletion of NAD^+^, whereas repletion with precursors, such as nicotinamide riboside, has shown a potential to reverse this process in experimental models [11,12,13]. NAD^+^ repletion through nicotinamide riboside oral supplementation in both young and aged mice has indeed shown the potential to increase muscle stem cell activity and regenerative capacity and to enhance muscle strength and performance in the context of aging [14]. Interestingly, treatment with nicotinamide riboside seems to also enhance muscle mitochondrial metabolism and exercise performance in mice in the context of metabolic dysfunction [12]. The NAD^+^ salvage pathway is partially impaired in sarcopenia since two rate-limiting enzymes are decreased in this condition, but de novo and exogenous pathways are intact and can likely be leveraged for therapeutic interventions. Nicotinamide riboside has been shown to be safe and efficacious to increase NAD^+^ levels in human blood and muscle in the context of aging [15,16,17], and there are early signals that it could contribute to muscle performance [18].

## 4. Urolithin A as an Emerging Nutritional Intervention for AACD (Dr. Anurag Singh)

**Figure 2:**
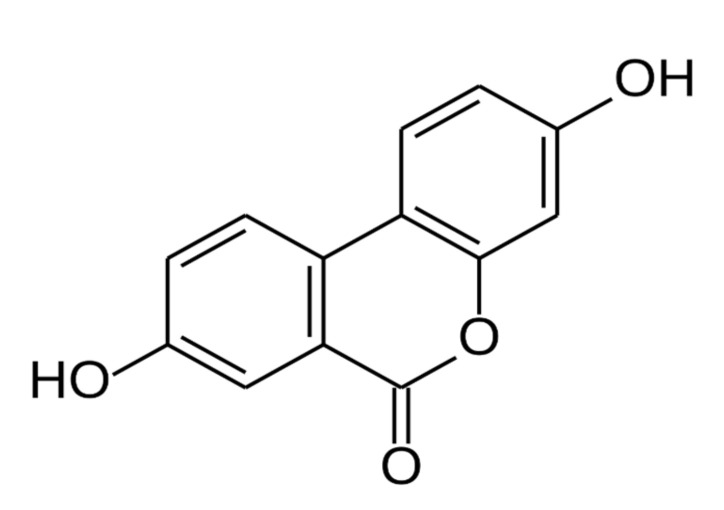
Chemical structure of urolithin A.

In humans, urolithins have been shown to be end-products or gut microbial metabolites of both ellagitannins and ellagic acid, which are complex polyphenols naturally contained in walnuts and fruits, such as pomegranates and red berries [19]. Urolithins A–D is measurable metabolites that have been identified in human plasma following consumption of ellagitannin- and/or ellagic acid-rich foods or extracts, with urolithin A being the predominant isoform (Figure 2) [20].

Ellagitannins are first hydrolyzed under the acidic conditions of the stomach into ellagic acid, which is then further metabolized by the microflora into urolithins via loss of one of its two lactones and by successful removal of hydroxyl groups [21]. However, the intestinal biotransformation rate of ellagic acid into urolithins varies substantially among individuals due to differences in gut microbiome composition [22]. Urolithin A has been shown to cross the basolateral membrane and has been identified in tissues of the colon, kidney, and liver in rodent models [23]. Once present in the bloodstream, urolithins undergo further metabolism by the liver as glucuronidated and sulfated metabolites. Bioavailability in skeletal muscle tissue has only recently been reported in humans [24].

With regard to safety, two rodent toxicological studies have evaluated the effects of high-dose ellagitannin supplementation at levels up to 4800 mg/kg of punicalagin (the most abundant ellagitannin in pomegranate) per day [25,26]. The safety and toxicological profile of both oral and intravenous synthetic, radiolabeled urolithin A administration also have been tested recently using a rodent model. Results from this study indicate an ingested no observed adverse effect with levels of 3451 and 3826 mg/kg per day in males and females over a 90-day period, respectively [27]. Urolithin A does not seem to demonstrate any genotoxic potential after repeated oral dosing, as shown by its null influence on hematological markers [27]. In the United States, urolithin A has been determined to be generally recognized as safe as an ingredient in food [28] and is, therefore, a legal dietary supplement in the United States per Section 201(ff) (2)-(3) of the Dietary supplement Health and Education Act of 1994 [29,30].

Research investigating the mechanism of action in regard to potential effects of urolithins on mitochondrial function and sarcopenia following dietary supplementation is emerging. In older worms (*Caenorhabditis elegans*), exposure to urolithin A activated mitochondrial biogenesis [31]. Mammalian C2C12 myoblasts and Mode-K intestinal cells treated with urolithin A showed dose-dependent elevation of biomarkers for both autophagy and mitophagy [31]. Chronic supplementation with urolithin A in mice fed a high-fat diet has been shown to increase muscle function measured at age 22 and 24 months. A subsequent rodent study showed supplementation to enhance average running endurance by 42% [31].

A single phase I (two-part) clinical study of urolithin A was reported in the scientific literature [32]. Part 1 of this study involved supplementing with single ascending doses of urolithin A (250–2000 mg) versus placebo in healthy elderly volunteers. The study was powered to provide sufficient information on the safety and pharmacokinetic profile of urolithin A and to allow for dose selection for future phase II efficacy trials. Part 2 consisted of supplementing multiple ascending doses of urolithin A (250, 500, and 1000 mg) versus placebo, each for 28 days. No product-related adverse events were reported at any dose of urolithin A in both parts of the study, and supplementation increased circulating levels of urolithin A in a dose-dependent manner. Several surrogate markers of mitochondrial health (e.g., mitochondrial respiratory complexes I, II, and IV abundance in *vastus lateralis*, mitochondrial respiratory complexes I, II, and IV activity in *vastus lateralis,* and relative abundance of mitochondrial DNA over nuclear DNA) were also investigated from skeletal muscle biopsies and blood samples at baseline and after 28 days of each dosing regimen. Dosing of urolithin A at 250 mg showed no significant improvement in biomarkers; however, dose-dependent decreases in acylcarnitine levels were demonstrated in the 500- and 1000-mg groups [24]. Acylcarnitines are the form in which fatty acids enter into the mitochondria to undergo oxidation. Urolithin A showed the most impact on shorter-chain acylcarnitines (C8, C10, C12, C14:1), which signifies better efficiency in the fatty acid oxidation process [33]. The direct impact of 500 and 1000 mg of urolithin A was shown through a dose-dependent upregulation of skeletal muscle mitochondrial gene expression in the human vastus lateralis. mRNA levels of autophagy/mitophagy and mitochondrial biogenesis-related biomarkers increased, as measured by quantitative PCR in the vastus lateralis, with some reaching statistical significance (GABA type A receptor-associated protein-like *GABARAPL1*, fatty acid-binding protein *FABP3*) [24]. These results were confirmed via transcriptomic profiling in the skeletal muscle that demonstrated significant upregulation of multiple mitochondrial-linked gene sets in the 500- and 1000-mg urolithin A groups compared to placebo.

## 5. Discussion

The scientific literature clearly demonstrates that ubiquitous cellular changes drive many age-associated chronic disease states. Targeting these specific pathways may have the potential to effectively alter the aging trajectory. Certain nutrients, including nicotinamide riboside and urolithin A, have been identified as promising ways to improve mitochondrial health, with each targeting different cellular pathways. Although the typical diet may provide low amounts of these nutrients, insufficient intakes are likely obtained due to their low abundance in commonly consumed foods and interindividual differences in their metabolization by the gut microbiome. Supplemental nicotinamide riboside has been shown to effectively boost NAD^+^ levels, which is important for mitochondrial biogenesis. Recent clinical data demonstrate urolithin A’s ability to activate cellular mitophagy and improve mitochondrial health in the skeletal muscle of healthy older adults. Additional research is underway on both of these cellular nutrients to understand their potential to impact cellular and clinical outcomes during aging.

## Figures and Tables

**Table 1:** Prioritized physical and environmental risk factors for age-associated cellular decline (AACD) (in rank order determined by the interdisciplinary expert panel).

Demographic and Clinical Risk Factors
Clinical conditions (e.g., cardiovascular disease)
Obesity
Unfavorable genetic background
Insulin resistance
Low physical capacity (e.g., slow gait speed and muscle weakness)
**Environmental and behavioral risk factors**
Smoking
Sedentary lifestyle
Low physical activity
Persistent physical or psychological stress
Low socioeconomic status
Alcohol abuse
Inadequate nutrition
Air pollution

Adapted with permission from Cesari et al. [4].

**Table 2:** Framework connecting biological mechanisms with the clinical phenotype of AACD.

Underlying Cellular and Subcellular Networks	Domains	Clinical Indicators
Cellular senescence	Energy metabolism	Fatigue
Mitochondrial abnormalities	Immune system	Low-quality of sleep
Metabolic signaling	Central nervous system	Low mood
Inflammation	Body composition	Lack of motivation
Autophagy/mitophagy		Subjective memory complaints
Oxidant/antioxidant balance		Poor exercise tolerance

Adapted with permission from Cesari et al. [4].

## Data Availability

No new data were created or analyzed in this study. Data sharing not applicable to this article.
